# Comparison of two swept-source optical coherence tomography biometers and a partial coherence interferometer

**DOI:** 10.1371/journal.pone.0223114

**Published:** 2019-10-11

**Authors:** Chan Min Yang, Dong Hui Lim, Hyo Jeong Kim, Tae-Young Chung

**Affiliations:** 1 Department of Ophthalmology, Samsung Medical Center, Sungkyunkwan University School of Medicine, Seoul, South Korea; 2 Department of Preventive Medicine, Graduate School, The Catholic University of Korea, Seoul, South Korea; Nicolaus Copernicus University, POLAND

## Abstract

**Purpose:**

To compare biometry and prediction of postoperative refractive outcomes obtained by two swept-source optical coherence tomography (SS-OCT) biometers (IOLMaster 700 and Argos), and a partial coherence interferometry (IOLMaster ver 5.4)

**Methods:**

Biometric values were measured using two SS-OCT and PCI device and evaluated against one another. Predictive errors were compared at one month after cataract surgery.

**Results:**

One hundred forty six eyes were considered. Axial length (AXL) measurements were not successful in 3 eyes measured by IOLMaster 700 and Argos devices, and in 17 eyes measured by IOLMaster ver. 5.4 devices. AXL as measured by Argos showed a tendency to be shorter in long eyes with AXL more than 26.0 mm (p < .001) and to be longer in short eyes with AXL less than 22.5 mm (p = .005). Anterior chamber depth as measured by IOLMaster ver. 5.4 was longer than that measured by the other two SS-OCT devices (vs. IOLMaster 700: p = .003; vs. Argos: p = .006). White-to-white diameter measured using Argos was significantly different measurements obtained using two IOLMaster (p < .001, respectively). The mean absolute postoperative prediction errors were 0.41 ± 0.31 diopters (D), 0.42 ± 0.32 D, and 0.35 ± 0.30 D for IOLMaster ver. 5.4, IOLMaster 700, and Argos, respectively.

**Conclusion:**

The ocular biometric measurements using three devices showed high agreement. AXL measured by Argos showed a significant difference compared with the measurements from two IOLMaster. ACD was highly correlated between two SS-OCT devices except IOLMaster ver 5.4. LT and CCT values between IOLMaster 700 and Argos were different significantly. SS-OCT devices demonstrated a superior ability to successfully perform measurements compared with PCI device.

## Introduction

Patient satisfaction after cataract surgery is influenced by the precise prediction of refractive outcomes and their subsequent realization. Accurate intraocular lens (IOL) power enables precise prediction of postoperative refractive status. In determining the IOL power, axial length (AXL), anterior chamber depth (ACD), lens thickness (LT), white-to-white corneal diameter (WTW) and keratometric value as well as a suitable IOL calculation formula, and IOL constant are required [1]. Accurate IOL power requires precise biometrics, in which AXL plays a major role [2]. Until recently, partial coherence interferometry (PCI) device such as IOLMaster (Carl Zeiss Meditec AG, Jena, Germany) have been the most frequently used one for measuring AXL and for performing IOL power calculations. However, AXL measurements can be difficult to obtain in individuals with mature cataract or severe posterior subcapsular (PSC) cataract using PCI-based device. Recently, a new type of device for biometry, which employs the principle of swept-source optical coherence tomography (SS-OCT) has been developed. SS-OCT-based devices use a longer wavelength light source than that used by conventional PCI devices. This can lead to an increase in the success rate of AXL measurement. Two- or three-dimensional OCT images obtained with these devices also show irregular eye geometries such as lens tilt. In addition, the fovea image can be used to identify insufficient patient fixation and reduce AXL error. The IOLMaster 700 700 (Carl Zeiss Meditec AG, Jena, Germany) is the first SS-OCT-based biometric device. Recently, though, another device based on the concept of SS-OCT, Argos (Movu, Inc., CA, U.S), OA-2000 (Tomey, Nagoya, Japan) Eyestar 900 (Haag Streit, Koeniz, Switzerland) were also introduced. Several studies have shown that the IOLMaster 700 has an acceptable degree of correlation of ability with proven PCI and optical low-coherence reflectometry (OLCR) device and a higher success rate of AXL measurement [3–5]. Shammas et al. also reported that measurements obtained with Argos were comparable to those made with PCI and OLCR devices but with a higher AXL acquisition rate [6]. However, there has been no study that has compared the aforementioned SS-OCT biometers.

As such, the purpose of current study was to compare the two SS-OCT-based devices and a conventional PCI device in terms of ocular biometry, success rate of AXL measurement, and prediction of postoperative refractive outcomes.

## Material and methods

This study was conducted involving patients who underwent ocular biometric measurements with IOLMaster ver 5.4, IOLMaster 700, and Argos in preparation to undergo cataract surgery at Samsung Medical Center, Seoul, Korea, between January 2017 and May 2017. This retrospective study was approved by the Institutional Review Board, Samsung Medical Center, Seoul, Korea. All work adhered to the tenets of the Declaration of Helsinki. The data included this work was analyzed anonymously. The patients’ medical records were reviewed, and patients with previous ocular trauma, those who had undergone prior refractive surgery, and those with corneal opacity, or corneal or another disease that affects visual acuity except cataract were excluded. All eyes with intraoperative complications such as capsular tear or posterior capsular rupture were also excluded.

One hundred forty-six eyes of 83 patients who met the inclusion criteria were evaluated. The mean patient age was 64.23 ± 10.51 years and 50 (60.24%) patients were female. Each eye included in this study was evaluated on the same day using the three devices, IOLMaster ver 5.4, IOLMaster 700, and Argos. Three examiners for each device performed the measurements in a random order. The measurements were performed before pupil dilatation in accordance with the respective manufacturers’ guideline.

### Instruments

IOLMaster ver. 5.4 uses PCI principle for measuring AXL. The keratometry value is measured by a six-point telecentric technique, while ACD via measured by lateral slit beam illumination. The IOLMaster ver. 5.4 can also measure the horizontal white-to-white corneal diameter (WTW) using LED light source. However, unlike with the two SS-OCT devices, LT and central corneal thickness (CCT) cannot measure by IOLMaster ver. 5.4.

IOLMaster 700 uses its SS-OCT scanning capacity with 1,050 nm laser infrared light and measures AXL, ACD, LT, and CCT. Like PCI device, IOLMaster 700 uses telecentric keratometry for keratometry measurements. WTW is measured in the same way by IOLMaster 700 as with PCI device.

Both IOLMaster devices, IOLMaster ver. 5.4 and IOLMaster 700, use a refractive index of 1.3375 for the biometry parameter.

Argos uses a 1,060 nm wavelength swept-source technology and records a two-dimensional OCT image from the corneal apex to the macula. With this OCT image, AXL, ACD, LT, and CCT can be measured. Standard refractive indices of 1.376 for the cornea, 1.336 for the aqueous and vitreous, and 1.410 for the lens are used to convert the optical diameter for measuring geometric distance. Keratometry is measured from the OCT image in conjunction with ring LED using a 1.3375 corneal index of refraction.

The IOL power calculated by each of the three device using Haigis [7] formula was obtained through medical chart review. Among the eligible patients for this retrospective study, we chose patients who were implanted with Acrysof SN60WF IOL (Alcon Laboratories, Inc. Fort Worth, TX, USA) to undergo evaluation of postoperative prediction error. For calculating the prediction error in IOL power calculation, we assessed the difference between the measured manifest refraction spherical equivalent at one month postoperation and the predicted refraction based on IOL power implanted in the patients’ eyes. The mean arithmetic prediction error (ME), mean absolute prediction error (MAE), median absolute prediction error (MedAE) and percentage of eyes with a prediction error within ± 0.5 diopters (D) were determined [8].

### Statistical analysis

Statistical analysis was performed using SPSS software ver. 22.0 (SPSS Inc., Chicago, IL, USA) and Excel software (Microsoft Corp, Redmond, WA, USA). The measurements were compared using paired t-test and Wilcoxon signed-rank test. Before data calculations, the normality was assessed using the Kolmogorov-Smirov test. Percentage of eyes with a prediction error within ± 0.5 D were analyzed with Chi-square test. Agreement between devices was analyzed using a Bland-Altman plot. The 95% limit of agreement (LoA) was defined as the mean ± 1.96 standard deviation of the difference between paired devices. The Pearson correlation coefficient (r) was used to evaluate the interdevice correlation. A p value less than 0.05 was considered statistically significant.

## Results

The success rate of AXL measurements for IOL master was 88.4% (129/146 eyes) and measurements of 17 eyes could not be obtained [three with mature white cataract, one with brunescent cataract, four with anterior subcapsular opacity(ASC), and nine with PSC)]. With the two SS-OCT devices (IOLMaster 700 and Argos), the AXL could be obtained in 143 (97.9%) of 146 eyes. Three eyes with mature white cataract were not able to undergo measurement of AXL by two SS-OCT devices.

### Comparison

Table 1 shows all of the biometric parameters as measured by the three devices.

**Table 1:** Summary of the comparison of values measured by the three devices.

Parameter	Device	Mean ± SD	Mean difference	SD of mean difference	p value	95% LoA	Pearson correlation coefficient (p value)
AXL (mm)	IOLMaster ver. 5.4	24.22 ± 1.96	-0.004	0.039	0.162 ^a^	-0.081 to +0.072	0.999 (<0.001)
	IOLMaster 700	24.22 ± 1.96					
	IOLMaster ver. 5.4	24.22 ± 1.96	0.026	0.070	<0.001 ^a^	-0.111 to +0.163	0.999 (<0.001)
	Argos	24.19 ± 1.92					
	IOLMaster 700	24.22 ± 1.96	0.030	0.057	<0.001 ^a^	-0.081 to 0.141	0.999 (<0.001)
	Argos	24.19 ± 1.92					
Kav (D)	IOLMaster ver. 5.4	44.01 ± 2.09	0.078	0.385	<0.001 ^a^	-0.467 to +0.665	0.983 (<0.001)
	IOLMaster 700	43.92 ± 2.14					
	IOLMaster ver. 5.4	44.01 ± 2.09	0.002	0.347	0.517 ^a^	-0.473 to +0.517	0.986 (<0.001)
	Argos	43.99 ± 2.10					
	IOLMaster 700	43.92 ± 2.14	-0.075	0.194	<0.001 ^a^	-0.456 to +0.303	0.996 (<0.001)
	Argos	43.99 ± 2.10					
ACD (mm)	IOLMaster ver. 5.4	3.20 ± 0.48	0.065	0.256	0.003 ^a^	-0.437 to +0.567	0.849 (<0.001)
	IOLMaster 700	3.13 ± 0.45					
	IOLMaster ver. 5.4	3.20 ± 0.48	0.061	0.263	0.013 ^a^	-0.455 to +0.576	0.841 (<0.001)
	Argos	3.14 ± 0.45					
	IOLMaster 700	3.13 ± 0.45	-0.004	0.066	0.444 ^a^	-0.133 to +0.125	0.989 (<0.001)
	Argos	3.14 ± 0.45					
WTW (mm)	IOLMaster ver. 5.4	11.58 ± 0.44	-0.150	0.312	<0.001 ^a^	-0.761 to +0.432	0.775 (<0.001)
	IOLMaster 700	11.73 ± 0.48					
	IOLMaster ver. 5.4	11.58 ± 0.44	-0.505	0.580	<0.001^b^	-1.641 to +0.631	0.554 (<0.001)
	Argos	12.08 ± 0.69					
	IOLMaster 700	11.73 ± 0.48	-0.355	0.563	<0.001^a^	-1.458 to +0.748	0.594 (<0.001)
	Argos	12.08 ± 0.69					
LT (mm)	IOLMaster 700	4.50 ± 0.60	-0.066	0.128	<0.001 ^a^	-0.317 to 0.184	0.977 (<0.001)
	Argos	4.56 ± 0.58					
CCT (μm)	IOLMaster 700	545.27 ± 37.58	24.048	9.877	<0.001 ^a^	+4.689 to +43.408	0.967 (<0.001)
	Argos	521.22 ± 39.02					

SD = standard deviation, LoA = limits of agreement, AXL = axial length, Kav = average keratometric value, ACD = anterior chamber depth, WTW = white-to-white distance, LT = lens thickness, CCT = central corneal thickness

^a^ Wilcoxon signed ranks test

^b^ Paired t-test

**Fig 1:**
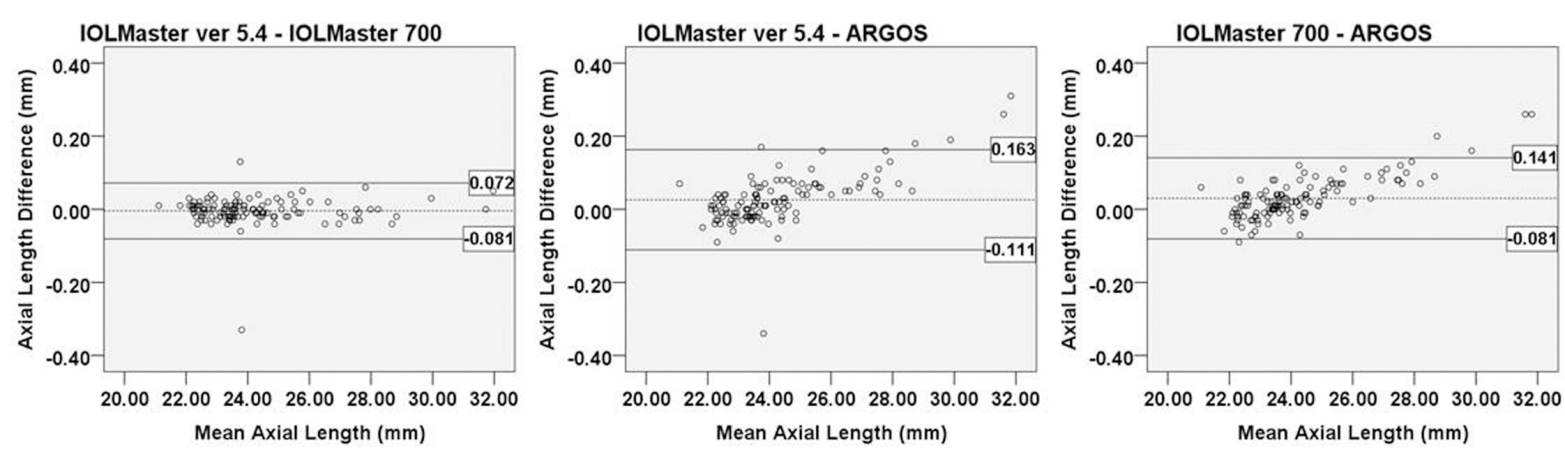
Bland-Altman plot of axial length measurements for each device. The mean difference is indicated by the dashed lines, and 95% LoA is indicated by the solid line. Comparison of IOLMaster ver. 5.4 and IOLMaster 700 (A); IOLMaster ver. 5.4 and Argos (B); and IOLMaster 700 and Argos (C).

The AXL was analyzed in all except 17 eyes, which could not be evaluated by IOLMaster ver. 5.4. The Pearson correlation coefficients of AXL were high (IOLMaster ver. 5.4 vs. IOLMaster 700: r = 0.999; IOLMaster ver. 5.4 vs. Argos: r = 0.999; IOLMaster 700 vs. Argos: r = 0.9996). The AXL measurements of IOLMaster ver. 5.4 and IOLMaster 700 were not statistically different (p = 0.162). However, Argos showed a statistically significant difference compared with the other two devices (p<0.001, respectively). In the Bland-Altman plot of AXL measurements, when the AXL was measured with Argos, the longer the AXL, the shorter the measured AXL. And the shorter the AXL, the longer the measured AXL. (Fig 1)

**Fig 2:**
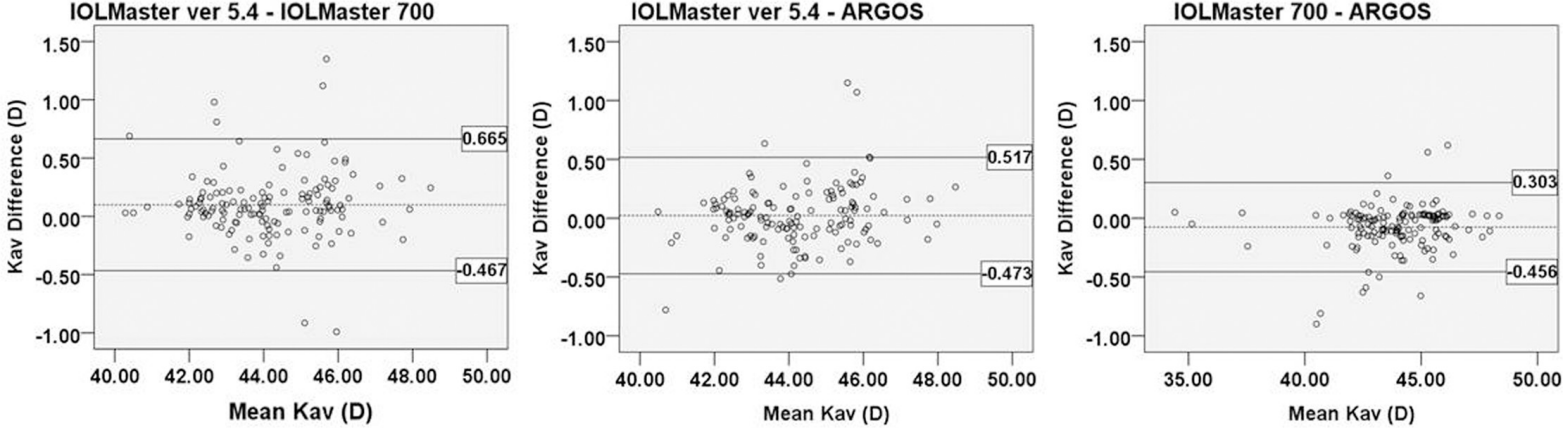
Bland-Altman plot of average keratometry measurements of each device. The mean difference is indicated by the dashed lines, and 95% LoA is indicated by the solid line. Comparison of IOLMaster ver. 5.4 and IOLMaster 700 (A); IOLMaster ver. 5.4 and Argos (B); and IOLMaster 700 and ARGOS (C). (Kav = average of keratometric value).

In the measurement of average keratometric value, the Pearson correlation coefficients were high (IOLMaster ver 5.4 vs. IOLMaster 700: r = 0.983, IOLMaster ver. 5.4 vs. Argos: r = 0.986; IOLMaster 700 vs. Argos: r = 0.996). IOLMaster 700 showed significantly flatter average keratometry measurements than did the other two devices (IOLMaster 700 vs. IOLMaster ver. 5.4: p = 0.017; IOLMaster 700 vs. Argos: p<0.001). However, the differences were small and clinically insignificant. The Bland-Altman plot showed good agreement with a narrow 95% LoA (Fig 2)

**Fig 3:**
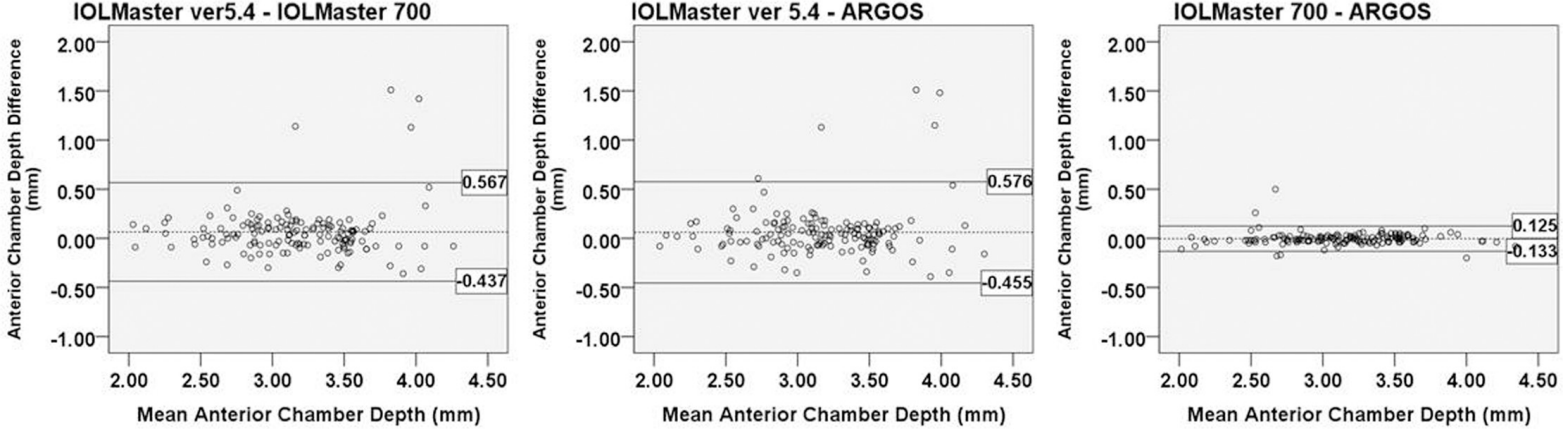
Bland-Altman plot for the ACD measurements of each device. The mean difference is indicated by the dashed lines, and 95% LoA is indicated by the solid line. Comparison of IOLMaster ver. 5.4 and IOLMaster 700 (A); IOLMaster ver. 5.4 and Argos (B); and IOLMaster 700 and Argos (C).

For comparison of ACD measurements, Pearson correlation coefficients were high (IOLMaster ver. 5.4 vs. IOLMaster 700: r = 0.849; IOLMaster ver. 5.4 vs. Argos: r = 0.841; IOLMaster 700 vs. Argos: r = 0.989). IOLMaster ver. 5.4 showed significantly longer ACD measurements than did the two SS-OCT devices (IOLMaster ver. 5.4 vs. IOLMaster 700: p = 0.003; IOLMaster ver. 5.4 vs. Argos: p = 0.006). The Bland-Altman plot showed good agreement with narrow 95% LoA (Fig 3). Especially, the agreement between IOLMaster 700 and Argos was extremely small (-0.133 to 0.125 mm).

**Fig 4:**
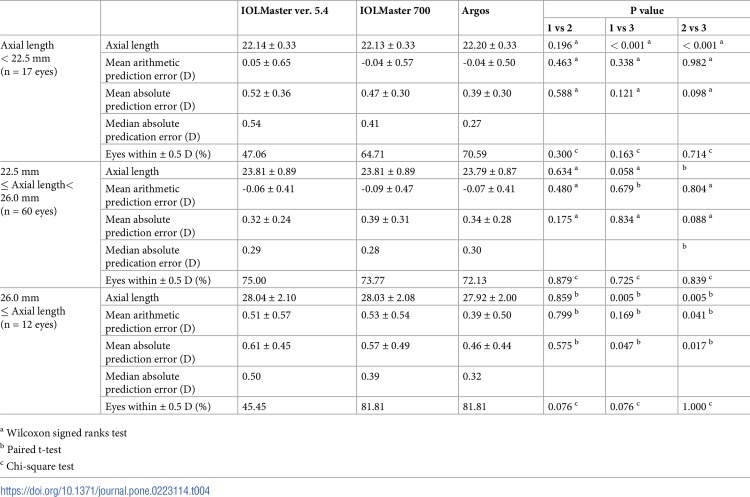
Bland-Altman plot for the WTW measurements of each devices. The mean difference is indicated by the dashed lines, and 95% LoA is indicated by the solid line. Comparison of IOLMaster ver. 5.4 and IOLMaster 700 (A); IOLMaster ver. 5.4 and Argos (B); and IOLMaster 700 and ARGOS (C).

In comparison of WTW, Argos showed significantly longer than did the other two devices (Argos vs. IOLMaster ver 5.4: p<0.001; Argos vs. IOLMaster 700: p<0.001). Pearson correlation coefficients between two IOLMaster devices were strongly positive (r = 0.775). However, Pearson correlation coefficients between Argos and two IOLMaster devices were of moderately positive strength (Argos vs. IOLMaster ver. 5.4: r = 0.554, p<0.001; Argos vs. IOLMaster 700: r = 0.594, p<0.001). The Bland-Altman plot of WTW is shown in Fig 4.

**Fig 5:**
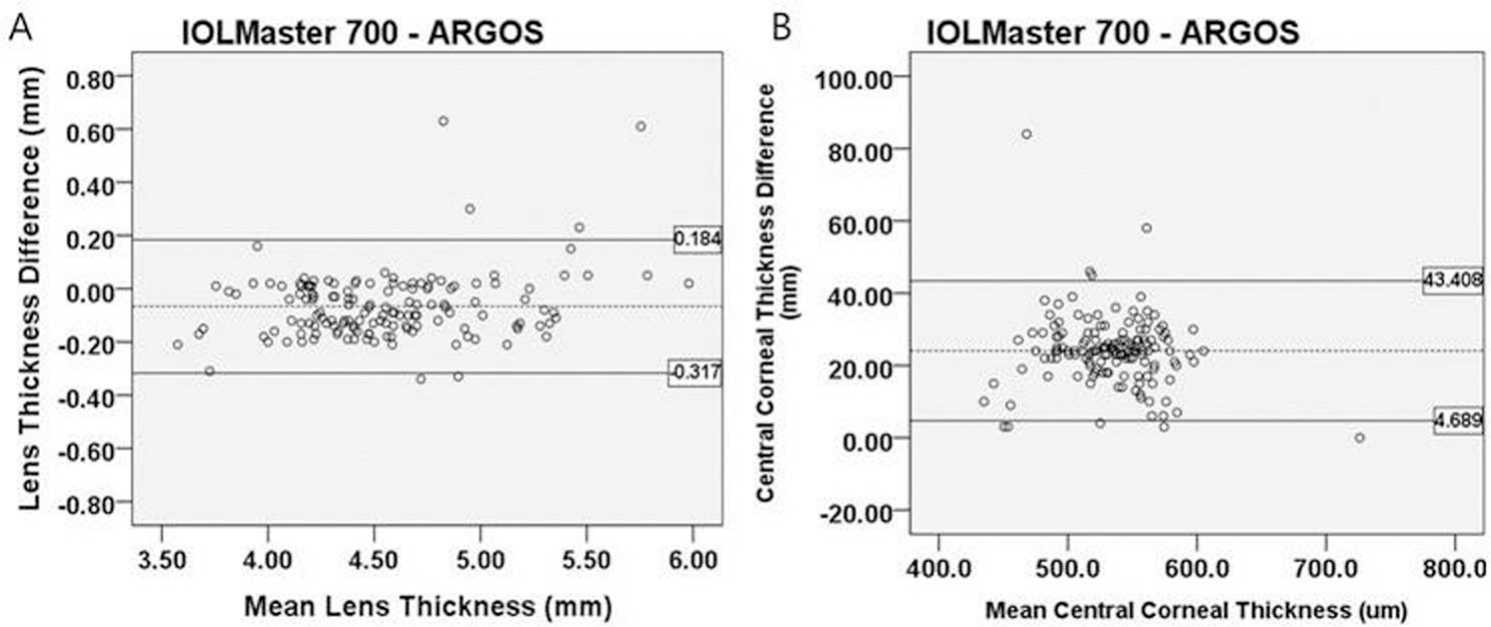
Bland-Altman plot for the LT and CCT measured by two SS-OCT devices. The mean difference is indicated by the dashed lines, and 95% LoA is indicated by the solid line. LT (A); CCT (B).

For LT and CCT, the Pearson correlation coefficients were 0.977 and 0.967, respectively. The relevant Bland-Altman plot is shown in Fig 5. LT and CCT values were statistically different between IOLMaster 700 and Argos (p<0.001, respectively)

### Postoperative refractive outcome

Among 106 eyes implanted with an Acrysof SN60WF IOL (Alcon Laboratories, Inc., Fort Worth, TX, USA), postoperative prediction errors were measured in 89 eyes in which AXL was measured by all three devices.

Table 2 shows the ME, MAE and percentage of eyes with a prediction error equal to or less than ± 0.5 D. The ME of IOLMaster ver. 5.4, IOLMaster 700, and Argos were 0.03 ± 0.51 D, -0.01 ± 0.53 D, and -0.01 ± 0.46 D, respectively, according to Haigis formula and showed no statistically significant difference (IOLMaster ver. 5.4 vs. IOLMaster 700: p = 0.177; IOLMaster ver. 5.4 vs Argos: p = 0.140; IOLMaster 700 vs. Argos: 0.724). The MAE of IOLMaster ver. 5.4, IOLMaster 700, and Argos were 0.41 ± 0.31 D, 0.42 ± 0.32 D, and 0.35 ± 0.30 D, respectively, when using the Haigis formula. Argos showed a significant difference in MAE compared with the two IOLMaster devices (IOLMaster ver. 5.4 vs. Argos: p = 0.043; IOLMaster 700 vs. Argos: p = 0.001). The percentages of eyes within ± 0.5 D were not statistically significantly different among the three devices (all p>0.05).

**Table 2:** Comparison of postoperative refractive errors between the three devices.

	IOLMaster ver. 5.4	IOLMaster 700	Argos	P value		
				1 vs 2	1 vs 3	2 vs 3
Mean arithmetic prediction error (D)	0.03 ± 0.51	-0.01 ± 0.53	-0.01 ± 0.47	0.177 ^a^	0.140 ^b^	0.735 ^a^
Mean absolute prediction error (D)	0.41 ± 0.31	0.42 ± 0.33	0.35 ± 0.30	0.866 ^a^	0.049 ^b^	0.001 ^a^
Median absolute predication error (D)	0.36	0.35	0.29	-	-	-
Eyes within ± 0.5 D (%)	68.54	73.03	73.03	0.510 ^c^	0.510 ^c^	0.999 ^c^

D = diopter

^a^ Wilcoxon signed ranks test

^b^ Paired t-test

^c^ Chi-square test

Table 3 shows the refractive errors among 14 eyes in which AXL was measured by only the two SS-OCT devices. The ME and MAE were within ± 0.5 D. The MEs of IOLMaster 700 and Argos were 0.10 ± 0.45 D and 0.15 ± 0.37 D, respectively (p = 0.453). The MAEs of IOLMaster 700 and Argos were 0.33 ± 0.32 D and 0.33 ± 0.22 D, respectively (p = 0.984). The refractive outcome of the 14 eyes was a slightly hyperopic shift.

**Table 3:** Postoperative arithmetic and absolute refractive errors of the two SS-OCT devices (n = 14) in eyes which AXL was not measured by the PCI device.

	IOLMaster 700	Argos	p value
Mean arithmetic prediction error (D)	0.10 ± 0.45	0.15 ± 0.37	0.453 ^a^
Mean absolute prediction error (D)	0.33 ± 0.32	0.34 ± 0.22	0.984 ^a^
Median absolute predication error (D)	0.22	0.28	-
Eyes within ± 0.5 D (%)	78.57	71.43	0.668 ^b^

^a^ Paired t-test

^b^ Chi-square test

Table 4 shows the ME, MAE and percentage of eyes with a prediction error equal to or less than ± 0.5 D according to AXL range. In eyes with an AXL shorter than 22.5 mm, AXL as measured by Argos was significantly longer than those measured by the two IOLMaster devices (p<0.001, respectively). The percentages of eyes within ± 0.5 D were not significantly different among the three devices (all p>0.05). The refractive outcomes also showed no significant differences among eyes with AXL shorter than 22.5mm, and AXL measurements were not significantly different between devices in eyes with AXL between 22.5 mm and 26.0 mm (all p>0.05). There were no significant differences in prediction errors in eyes with AXL between 22.5 mm and 26.0 mm. The percentages of eyes within ± 0.5 D were not statistically significantly different among the devices (all p>0.05). In eyes with AXL longer than 26.0 mm, AXL as measured by Argos was statistically significantly shorter than those measured by two IOLMaster devices (IOLMaster ver. 5.4 vs. ARGOS p = 0.005; IOLMaster 700 vs. Argos: p = 0.005). Argos showed a significant difference compared with two IOLMaster devices (IOLMaster ver. 5.4 vs. Argos: p = 0.047; IOLMaster 700 vs. Argos: p = 0.017). Two SS-OCT devices provided better results than PCI device with respect to the percentage of eyes within ± 0.5 D with AXL longer than 26.0mm.

**Table 4:** Comparison of postoperative refractive errors according to axial length range.

		IOLMaster ver. 5.4	IOLMaster 700	Argos	P value		
					1 vs 2	1 vs 3	2 vs 3
Axial length < 22.5 mm (n = 17 eyes)	Axial length	22.14 ± 0.33	22.13 ± 0.33	22.20 ± 0.33	0.196 ^a^	< 0.001 ^a^	< 0.001 ^a^
	Mean arithmetic prediction error (D)	0.05 ± 0.65	-0.04 ± 0.57	-0.04 ± 0.50	0.463 ^a^	0.338 ^a^	0.982 ^a^
	Mean absolute prediction error (D)	0.52 ± 0.36	0.47 ± 0.30	0.39 ± 0.30	0.588 ^a^	0.121 ^a^	0.098 ^a^
	Median absolute predication error (D)	0.54	0.41	0.27			
	Eyes within ± 0.5 D (%)	47.06	64.71	70.59	0.300 ^c^	0.163 ^c^	0.714 ^c^
22.5 mm ≤ Axial length< 26.0 mm (n = 60 eyes)	Axial length	23.81 ± 0.89	23.81 ± 0.89	23.79 ± 0.87	0.634 ^a^	0.058 ^a^	0.062 ^a^
	Mean arithmetic prediction error (D)	-0.06 ± 0.41	-0.09 ± 0.47	-0.07 ± 0.41	0.480 ^a^	0.679 ^b^	0.804 ^a^
	Mean absolute prediction error (D)	0.32 ± 0.24	0.39 ± 0.31	0.34 ± 0.28	0.175 ^a^	0.834 ^a^	0.088 ^a^
	Median absolute predication error (D)	0.29	0.28	0.30			
	Eyes within ± 0.5 D (%)	75.00	73.77	72.13	0.879 ^c^	0.725 ^c^	0.839 ^c^
26.0 mm ≤ Axial length (n = 12 eyes)	Axial length	28.04 ± 2.10	28.03 ± 2.08	27.92 ± 2.00	0.859 ^b^	0.005 ^b^	0.005 ^b^
	Mean arithmetic prediction error (D)	0.51 ± 0.57	0.53 ± 0.54	0.39 ± 0.50	0.799 ^b^	0.169 ^b^	0.041 ^b^
	Mean absolute prediction error (D)	0.61 ± 0.45	0.57 ± 0.49	0.46 ± 0.44	0.575 ^b^	0.047 ^b^	0.017 ^b^
	Median absolute prediction error (D)	0.50	0.39	0.32			
	Eyes within ± 0.5 D (%)	45.45	81.81	81.81	0.076 ^c^	0.076 ^c^	1.000 ^c^

^a^ Wilcoxon signed ranks test

^b^ Paired t-test

^c^ Chi-square test

## Discussion

Recently, cataract surgery has been aimed at also achieving more accurate refractive correction. Therefore, accurate ocular biometry has become important. Various instruments such as A-scan ultrasound biometry, PCI, OLCR, and SS-OCT devices are used for measurement of accurate biometrics. A few studies have specifically investigated the use of SS-OCT for this purpose. However, to our knowledge, there was no previous study comparing two SS-OCT devices divided into three groups according to axial length. In this study, we compared values of ocular biometry and the prediction of postoperative refractive outcomes as obtained by two SS-OCT devices and PCI device.

In the present study, 14 eyes had dense PSC, ASC and brunescent cataract that could be measured using two SS-OCT devices but not the PCI device. Using two SS-OCT devices (IOLMaster 700 and Argos), 143 (97.9%) of 146 eyes could be evaluated for AXL measurement compared with 129 (88.4%) with PCI device (IOLMaster ver. 5.4). Previous studies involving IOLMaster 700 showed a high success rate correlated with its use in the acquisition of AXL measurements (92.5% ~ 100%) [3–5, 9]. Shammas et al. also reported a high measurement success rate of 96% with Argos [6]. The majority of previous reports of PCI and OLCR acquisition success rates of AXL measurements report correlation with presence of mature cataract or PSC[10–12]. The difference in success rate of AXL measurements between SS-OCT devices and other measurement devices can be influenced by the differences among light sources and scanning patterns used. The reason why there is a higher success rate of AXL measurement with SS-OCT is probably because it can use longer wavelengths (IOLMaster 700: 1,050 nm Argos: 1,060 nm). The ability to use a longer wavelength than that used by PCI (780 nm) can reduce light scattering from opaque media, allowing greater penetration through a severe cataract.

In comparison of AXL, the differences between Argos and other devices were statistically significant. However, there was an additional difference according to AXL range. AXL measured with Argos was shorter in long eyes with AXL more than 26.0 mm and longer in short eyes with AXL less than 22.5 mm. Shammas et al. report that the AXL measurements by Argos had good agreement with those of PCI and OLCR devices [6]. However, their study did not report the difference in AXL measurement according to the AXL range for each device. Seventeen eyes with AXL less than 22.5 mm were included in the present study. In these short eyes, AXL measured by Argos was slightly longer than that measured by two IOLMaHowever, to our knowledge, there has been no previouster devices. AXL length measured by Argos in 12 eyes longer than 26.0 mm was shorter than that measured by two IOLMaster devices in all 12 eyes. Generally, longer eyes have a relatively larger proportion of vitreous in the total AXL, while shorter eyes have a relatively larger proportion of crystalline lens in the total AXL. In the normal AXL range, composite refractive index does not affect AXL measurement results, and our study also shows that the difference is not significant. However, in long or short eyes, it may be more accurate to apply a different refractive index to account for differences in the crystalline lens and the vitreous. Argos calculated AXL as the sum of physical distances of four segments; CCT, aqueous depth, LT, and the thickness of the vitreous to the retina which were each calculated by dividing the optical distance by the corresponding refractive index (1.374, 1.336, 1.41, and 1.336, respectively), which thus implies the true physical scale of AXL. However, the two IOLMaster devices use a composite refractive index (1.3549). Wang et al report that an additional adjustment to the AXL value as calculated by traditional biometer devices is required to take into account the offset from the true AXL value before substituting it into the IOL power formula in eyes with AXLs measuring more than 25.0 mm [13]. It seems that using a corresponding refractive index can more accurately measure AXL.

There was no statistically difference in average keratometric value between IOLMaster ver. 5.4 and Argos (p = 0.939). IOLMaster 700 results were flatter than those of the other two biometers. However, 95% LoA value of keratometic values are too broad, which may be clinically meaningful. Akman et al. and Yoo et al. also report similar results, in that keratometric value as measured by IOLMaster 700 was statistically significantly decreased compared with that measured by PCI devices [3, 9].

The ACD measurements of the two SS-OCT devices showed good agreement, with a very narrow 95% LoA. ACD measured by PCI device was significantly longer than that measured by the two SS-OCT devices (IOLMaster ver. 5.4 vs. IOLMaster 700: p = 0.003; IOLMaster ver. 5.4 vs. Argos: p = 0.006). Hoffer et al. and Villalobos et al. also report that the value measured by IOLMaster 700 was significantly smaller than those measured by PCI devices [4, 14]. The difference between the two SS-OCT devices and the PCI device might be due to difference in measurement technique for each type of device. SS-OCT measures ACD in the optical axis. However, PCI device may not measure the optical axis because this type of device uses a lateral slit beam technique. Because the fourth-generation IOL calculation formulas such as Holladay II and Haigis use ACD, SS-OCT seems to produce more accurate results.

**Fig 6:**
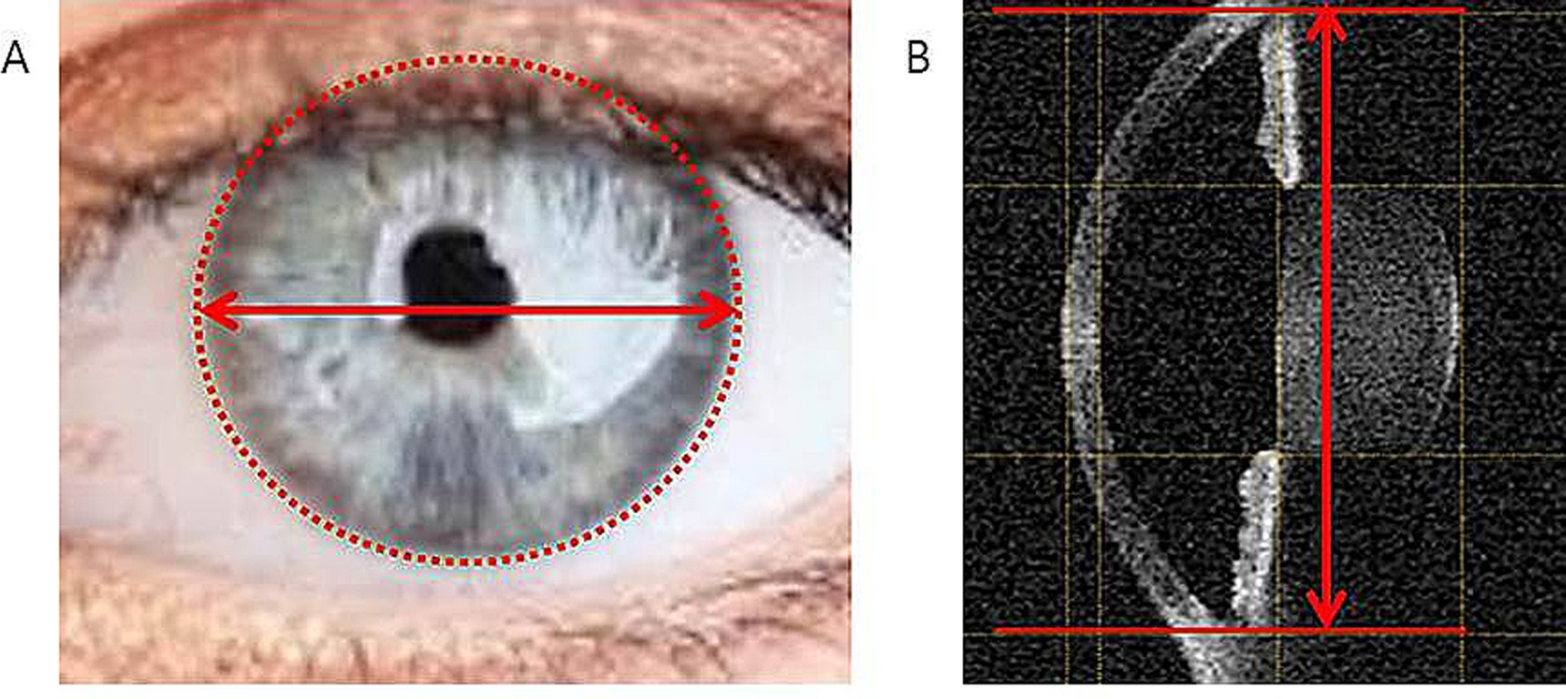
WTW measurements of each device. The two IOLMaster devices produce a camera image using LED light source and measure the diameter of a circle fitted to the corneal contour from the camera image (A). Argos tries to identify the junction of the posterior cornea and iris (B).

WTW measured by Argos was significantly longer than that measured by the two IOLMaster devices (Argos vs. IOLMaster ver. 5.4: p<0.001; Argos vs. IOLMaster 700: p<0.001). This difference may be attributed to the different measurement techniques of each device. While the two IOLMaster devices measure the diameter of the corneal contour in a camera-based image, Argos tries to identify the junction of the posterior cornea and iris in an OCT image (Fig 6). In Holladay II formula using WTW for calculation of IOL power, the refractive outcome between the two SS-OCT devices may be different.

The mean difference of the LT measured by the two SS-OCT devices was significant (p<0.001). However, the value was very small. Knunert et al. reported that the difference in LT measured with IOLMaster 700 and OLCR was 0.021 mm and was not clinically significant [15]. Our results showed that a larger difference between IOLMaster 700 and Argos than that seen in Knunert et al.

In a comparison of CCT, Argos found thinner results in all eyes and the mean difference was statistically significant (p<0.001). Hoffer et al. and Knuert et al. reported that the difference in CCT as measured by IOLMaster 700 and OLCR was very small (Hoffer et a.: 5 um; Knuert et al.: 0.15 um) [14, 15]. In this study, the mean difference of CCT value between the two SS-OCT devices was somewhat greater than that in other studies.

Most of the above-mentioned parameters have statistically significant differences for each device. However, it was a clinically very small difference in terms of numerical value. However, the average CCT measured by IOLMaster 700 and ARGOS differ by more than 20 micrometers. Although CCT is not vital in IOL calculation, it is important for accurate measurement of intraocular pressure in glaucoma and in preoperative assessment for laser refractive surgery. Therefore, IOLMaster 700 and Argos cannot be considered interchangeable for measuring CCT.

The ME and percentage of eyes within ± 0.5 D were no statistically significant difference among the three devices. The percentage of eyes within + 0.5D of the two SS-OCTs were about 5% higher than that of PCI device. It may be clinically meaningful if sample size was bigger. Argos was statistically significantly smaller than those of the two IOLMasters devices (IOLMaster ver. 5.4 vs. Argos: p = 0.043; IOLMaster 700 vs. Argos: p = 0.001). Additionally, there was also a statistically significant difference in long eyes with AXL more than 26.0 mm (IOLMaster ver. 5.4 vs. Argos: p = 0.047; IOLMaster 700 vs. Argos: p = 0.017). In eyes with AXL less than 22.5 mm, there was no statistically significant difference in MAE between the devices (IOLMaster ver. 5.4 vs. IOLMaster 700: p = 0.588; IOLMaster ver.5.4 vs. Argos: p = 0.121; IOLMaster 700 vs. Argos: p = 0.098). However, Argos produced a value more closer to emmetropia. There may also have been a meaningful difference if more short eyes were included in the analysis.

The MAE of the eyes not measured by conventional PCI but only measured by the two SS-OCT devices was less than 0.5 D. However, both SS-OCT devices showed slightly more hyperopic appearances than the preoperative target refraction. It seems that the AXL is measured to be slightly longer than actual. Since light travels slowly in dense cataract, it may be possible that the measured AXL is longer than the actual AXL and results in hyperopic shift. Therefore, it may be helpful to keep this in mind when evaluating such patient, and further studies should be considered involving patients with brunescent cataract or white intumescent cataract.

The main limitation of this study is its retrospective design. Our study does not contain many eyes with AXL longer than 26 mm or shorter than 22 mm. It is necessary to confirm the difference of AXL measurement between Argos and other devices and the change in refractive outcome according to measurements in long eyes and short eyes. Also, our study did not include enough patients with dense cataract and compare the results of other fourth-generation IOL formulas such as Holladay II, Barrett Universal II, Olsen, and Hill-RBF.

AXL measurement with Argos was slightly shorter than that with the two IOLMaster devices, especially in eyes with AXL more than 26.0 mm. Additionally, in short eyes, Argos yielded longer AXL measurement values than did the two IOLMaster devices. Although there were differences in WTW and LT measurements, there was no significant difference in refractive error after surgery. If the AXL is not measured using an existing PCI device, SS-OCT can be useful due to its high success rate of AXL measurement. In addition, SS-OCT-associated refractive prediction error is low, and the instrument is expected to be a useful tool for accurate refractive correction in patients with cataract.

## Supporting information

S1 Data Data analyzed. (XLSX)
